# Type of occupation and early antenatal care visit among women in sub-Saharan Africa

**DOI:** 10.1186/s12889-022-13306-6

**Published:** 2022-06-04

**Authors:** Abdul-Aziz Seidu, Edward Kwabena Ameyaw, Francis Sambah, Linus Baatiema, Joseph Kojo Oduro, Eugene Budu, Francis Appiah, Bright Opoku Ahinkorah

**Affiliations:** 1 Centre For Gender and Advocacy, Takoradi Technical University, Takoradi, Ghana; 2 Department of Estate Management, Faculty of Built and Natural Environment, Takoradi Technical University, Takoradi, Ghana; 3 College of Public Health, Medical and Veterinary Sciences, James Cook University, Townsville, QLD Australia; 4 School of Public Health, Faculty of Health, University of Technology Sydney, Sydney, Australia; 5 L & E Research Consult, Upper West Region Wa, Ghana; 6 Department of Health, Physical Education and Recreation, University of Cape Coast, Cape Coast, Ghana; 7 Department of Population and Health, University of Cape Coast, Cape Coast, Ghana; 8 Department of Social Sciences, Berekum College of Education, Berekum, Bono Region Ghana

**Keywords:** Early ANC, Occupation, Maternal health, Pregnancy, SSA, Public Health

## Abstract

**Background:**

Type of occupation has been linked to early antenatal care visits whereby women in different occupation categories tend to have different timing for antenatal care visits. Different occupations require varying levels of commitment, remuneration and energy requirements. This study, therefore, sought to investigate the association between the type of occupation and early antenatal care visits in sub-Saharan Africa.

**Methods:**

This is a secondary analysis of Demographic and Health Survey data from 29 countries in sub-Saharan Africa conducted between 2010 and 2018. The study included 131,912 working women. We employed binary logistic regression models to assess the association between type of occupation and timely initiation of antenatal care visits.

**Results:**

The overall prevalence of early initiation of antenatal care visits was 39.9%. Early antenatal care visit was high in Liberia (70.1%) but low in DR Congo (18.6%). We noted that compared to managerial workers, women in all other work categories had lower odds of early antenatal care visit and this was prominent among agricultural workers [aOR = 0.74, CI = 0.69, 0.79]. Women from Liberia [aOR = 3.14, CI = 2.84, 3.48] and Senegal [aOR = 2.55, CI = 2.31, 2.81] had higher tendency of early antenatal care visits compared with those from Angola.

**Conclusion:**

The findings bring to bear some essential elements worth considering to enhance early antenatal care visits within sub-Saharan Africa irrespective of the type of occupation. Women in the agricultural industry need much attention in order to bridge the early antenatal care visit gap between them and workers of other sectors. A critical review of the maternal health service delivery in DR Congo is needed considering the low rate of early antenatal care visits.

## Background

Sub-Saharan Africa (SSA) is one of the World Health Organization (WHO) regions with the highest maternal mortality ratio (MMR) worldwide [1]. Antenatal care (ANC) has been recognised as a promising strategy for averting threats that compromise the health of pregnant women and subsides MMR prospects [2, 3]. ANC is “the care provided by skilled health-care professionals to pregnant women and adolescent girls in order to ensure the best health conditions for both mother and baby during pregnancy” [4]. The WHO conceives early ANC as the initial visit occurring within the first 12 weeks of pregnancy [4]. Early ANC is recommended for all pregnant women irrespective of occupation, socio-economic status, geographical location, parity inter alia. Prevention and management of pregnancy-related diseases, risk identification, health education and health promotion are some of the core components of ANC [4, 5]. ANC reduces the likelihood of maternal and perinatal morbidity and mortality in two principal ways-by identifying and treating pregnancy-related complications and through identification of women who are at higher risk of labour and delivery complications [6–8].

Early ANC is observed to be low in SSA [9]. Globally, SSA is the penultimate region with lowest early ANC visit coverage (24·9% [22·6–27·2]) after Oceania [10]. Reports of recent Demographic and Health Surveys have revealed same. For instance, 18%, 20% and 37% of women are reported to have obtained early ANC in Nigeria [11], Ethiopia [12] and Zambia [13] respectively. A considerable section of women in SSA tend to delay and commence ANC in the second or third trimester [14]. It is known that education [15], younger age [16, 17], family income [18] and residential status [19, 20] dictate the timing of ANC.

Occupational type has also been linked with early ANC initiation whereby women who work in particular professions seem to have early ANC visits [14, 21]. This is believed to be enhanced by the relative economic advantage associated with particular occupations over others [9, 21]. Maternal health care is cost-free in a number of sub-Saharan African countries [22, 23]. Depending on a woman’s occupation type, some women seem to have relative economic advantage to pay the additional expenses such as those originating from transportation [22, 24] laboratory tests and screening [23] and unauthorised charges levied by some healthcare providers [25]. The aforementioned studies have principally investigated occupational status (working or not working) without exploring early ANC initiation across the type of occupation (such as services, agriculture and clergy).

Due to that, whether the driving and inhibition factors of early ANC visits vary across women’s occupations seem unexplored in SSA. Meanwhile, different occupations have different commitment levels, time requirements, remuneration, and energy requirements [26, 27]. This study, therefore, proposes that the type of occupation women engage in may have varying implications on their prospects of attaining early ANC visits. Investigating early ANC visits by type of occupation, as this study seeks to achieve, is of utmost priority for maternal health in order to develop pertinent demand-driven, well-tailored and fit for purpose interventions that can support all category of career women to achieve timely ANC visits in SSA and other developing WHO regions.

## Methods

### Study design and data source

This study analysed a secondary data from working women of reproductive ages (*n* = 131,192) who had complete information on ANC attendance from the latest Demographic and Health Surveys (DHS) conducted between 2010 and 2018 across 29 countries in SSA (see Table 1). The survey was designed to collect and provide data on various demographic indicators such as maternal healthcare services utilization [28]. The data collected through DHS are robust, helpful in health research and are used to study and monitor prevalence, pattern and trends of health information in low- and middle-income countries [29]. To select the sample, two multi-stage stratified cluster sampling methods were employed and the eligible respondents were selected from rural and urban areas in the various countries. Data were collected from women, men, couples and children by using different questionnaires. Standard methods such as the use of experienced field staff and validated instruments were employed to test the validity and reliability of the DHS questionnaires. The details of the DHS are documented by Corsi, Neuman, Finlay and Subramanian [30]. The datasets for the DHS are available at http://dhsprogram.com/data/available-datasets.cfm.

**Table 1:** Description of Study Sample

Country	Sample N	Sample %
Angola, 2015/2016	4830	3.7
Burkina Faso, 2010	8007	6.1
Benin, 2017/18	6562	5.0
Burundi, 2016/2017	8190	6.2
Congo DR, 2013/2014	7815	5.9
Congo, 2011/2012	3762	2.9
Cote d’Ivoire, 2011/2012	3446	2.6
Cameroon, 2018	2200	1.7
Ethiopia, 2016	2231	1.7
Gabon, 2012	1738	1.3
Ghana, 2014	3313	2.5
Gambia, 2013	2933	2.2
Guinea, 2018	3464	2.6
Kenya, 2014	4659	3.5
Comoros, 2012	786	0.6
Liberia, 2013	2721	2.1
Lesotho, 2014	1001	0.8
Mali, 2018	3234	2.5
Malawi, 2015/2016	9374	7.1
Nigeria, 2018	12599	9.6
Namibia, 2013	1610	1.2
Rwanda, 2014/2015	5570	4.2
Sierra Leone, 2013	6641	5.0
Senegal, 2010	4571	3.5
Chad, 2014/2015	1412	1.1
Togo, 2013/2014	3645	2.8
Uganda, 2016	8157	6.2
Zambia, 2018	5026	3.8
Zimbabwe, 2015	2416	1.8
**Total**	131912	100

### Derivation of variables

#### Outcome variable

The outcome variable of the study was early ANC attendance. It was derived from the question “How many months pregnant were you when you first received antenatal care for this pregnancy?” The responses were in months. It was then dichotomised as early initiation of ANC = 1, that is if women reported attending ANC at 3 months or earlier and late initiation = 0, after 3 months [31, 32].

#### Independent variable

Type of occupation was the independent variable [33]. It was generated from the question “﻿What is your occupation, that is, what kind of work do you mainly do?” Occupation was captured as ‘not working (0)’, ‘managerial (1)’, ‘clerical (2)’, ‘sales (3)’, ‘agricultural (4)’, ‘household (5)’, ‘services (6)’ and ‘manual (7)’. We excluded those who were not working to align the sample to the focus of the study.

#### Control variables

Fifteen control variables were considered in our study. These are country, age, educational level, marital status, religion, wealth quintile, place of residence, parity, pregnancy intention, exposure to mass media (radio, television and newspaper) and getting medical help for self (getting permission to go, getting money needed for treatment and distance to health facility). Apart from country of origin, the rest of the variables were not determined a priori; instead, based on parsimony, theoretical relevance and practical significance with early initiation of ANC [3, 19, 31, 32, 34]. Marital status was recoded into never married, married, cohabiting, widowed and divorced. We recoded parity as one birth (1), two births (2), three births (3), and four or more births (4). We recoded religion as Christianity (1), Islam (2), Traditionalist (3), and no religion (4).

### Statistical analyses

**Fig. 1:**
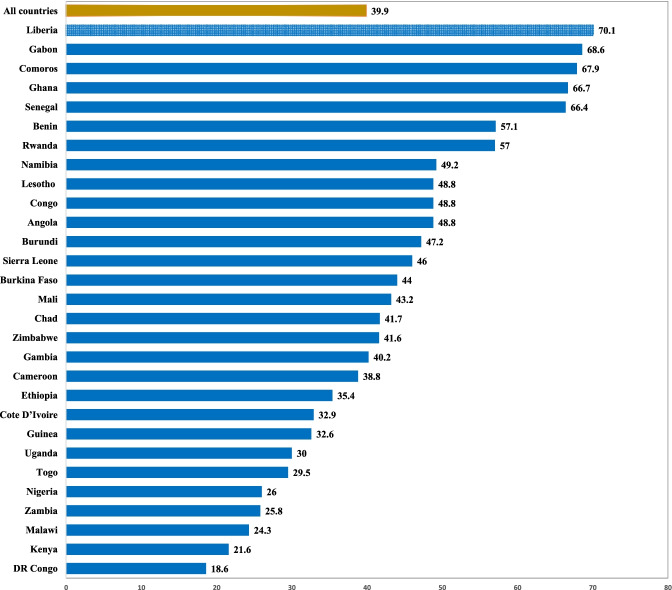
Prevalence of early initiation of antenatal care among working women in sub-Saharan Africa

The data were analysed with stata version 14.2 for Mac OS. The analysis was done in three steps. The first step was the computation of the prevalence of early initiation of ANC among working women in SSA (see Fig. 1). The second step was a bivariate analysis that calculated the prevalence of early initiation of ANC across the socio-demographic characteristics with their significance levels at p < 0. 05 (Table 2). Afterwards, two logistic regression models were built. Model I was a bivariate analysis between the key independent variable, women’s occupation, and early ANC attendance. Model II controlled for the effect of country and all the socio-demographic variables in a multivariable logistic regression (see Table 3). All frequency distributions were weighted while the survey command (svy) in stata was used to adjust for the complex sampling structure of the data in the regression analyses. Multicollinearity was checked and there was no evidence of multicollinearity among the variables (Max VIF = 2.06; mean VIF = 1.38). All results of the logistic analyses were presented as crude odds ratios (cORs for Model I) and adjusted odds ratios (aORs for Model II) with 95% confidence intervals (CIs).

**Table 2:** Type of occupation, socio-demographic characteristics and prevalence of early antenatal care visit among women in sub-Saharan Africa (n = 131,912)

**Variable (p value)**	**Weighted**	**Weighted**	**ANC attendance**	
	** N**	**%**	**Late (%)**	**Early (%)**
**Type of occupation (p<0.001)**				
Managerial	7183	5.5	47.0	53.0
Clerical	1072	0.8	51.6	48.4
Sales	37482	28.4	58.2	41.8
Agriculture	57992	44.0	63.1	36.9
Services	14319	10.9	60.3	39.7
Manual	13864	10.5	59.8	40.2
**Age (p<0.001)**				
15-19	7115	5.4	64.5	35.5
20-24	26207	19.9	61.1	38.9
25-29	34373	26.1	58.8	41.2
30-34	29015	22.0	59.2	40.8
35-39	21110	16.0	59.8	40.2
40-44	10567	8.0	61.0>	39.0
45-49	3524	2.7	63.0	37.0
**Level of Education (p<0.001)**				
No formal education	47046	35.7	60.7	39.3
Primary	46382	35.2	63.4	36.7
Secondary	32735	24.8	57.1	42.9
Higher	5749	4.4	45.9	54.1
**Marital status (p<0.001)**				
Never married	7739	5.9	56.3	43.7
Married	91058	69.0	58.4	41.7
Cohabitation	22532	17.1	55.1	44.9
Widowed	2151	1.6	60.4	39.6
Divorced	8432	6.4	57.2	42.8
**Religion (p<0.001)**				
Christianity	85694	65.0	60.7	39.3
Islam	39667	30.1	58.7	41.3
Traditional	3918	3.0	58.3	41.7
No religion	2633	2.0	65.7	34.3
**Wealth quintile (p<0.001)**				
Poorest	26421	20.0	64.9	35.1
Poorer	27723	21.0	63.9	36.1
Middle	27301	20.7	62.2	37.8
Richer	26620	20.2	58.8	41.3
Richest	23847	18.1	49.5	50.5
**Residence (p<0.001)**				
Urban	43696	33.1	54.1	45.9
Rural	88216	66.9	63.1	36.9
**Parity (p<0.001)**				
1	24389	18.5	55.5	44.5
2	24562	18.6	56.7	43.3
3	21597	16.4	58.4	41.6
4+	61364	46.5	63.9	36.1
**Pregnancy intentions (p<0.001)**				
Planned	94116	71.4	58.4	41.6
Mistimed	28311	21.5	63.7	36.3
Unwanted	9485	7.2	66.7	33.3
**Household head sex (p<0.001)**				
Male	103500	78.5	60.7	39.3
Female	28412	21.5	57.9	42.1
**Frequency of reading newspaper/magazine (p<0.001)**				
Not at all	110555	83.8	61.4	38.6
Less than once a week	11830	9.0	56.5	43.5
At least once a week	8629	6.5	50.7	49.3
Almost every day	898	0.7	36.6	63.4
**Frequency of watching television (p<0.001)**				
Not at all	80417	61.0	64.2	35.8
Less than once a week	16956	12.9	58.8	41.2
At least once a week	27283	20.7	52.7	47.3
Almost every day	7256	5.5	45.7	54.3
**Frequency of listening to radio (p<0.001)**				
Not at all	50640	38.4	64.3	35.7
Less than once a week	27152	20.6	58.7	41.3
At least once a week	49085	37.2	57.1	42.9
Almost every day	5036	3.8	55.7	44.3
**Getting medical help for self: getting permission to go (p<0.001)**				
No problem	4274	3.2	52.9	47.1
Big problem	20631	15.6	61.7	38.3
Not a big problem	107008	81.1	60.1	39.9
**Getting medical help for self: getting money needed for treatment (p<0.001)**				
No problem	893	0.7	55.6	44.4
Big problem	72486	55.0	61.6	38.4
Not a big problem	58533	44.4	58.4	41.7
**Getting medical help for self: distance to health facility** **(p<0.001)**				
No problem	2241	1.7	51.3	48.7
Big problem	49795	37.8	62.9	37.2
Not a big problem	79876	60.6	58.7	41.4

## Results

### Descriptive results

Figure 1 is a graphical presentation of early initiation of ANC among women in SSA. Early initiation of ANC among working women across SSA stood at 39.9%. However, early initiation of ANC was high among Liberian women (70.1%) and low among women in DR Congo (18.6%)﻿.

Table 2 displays results on type of occupation, socio-demographic characteristics and early ANC attendance among working women in SSA. It was found that a significant proportion of women who were into managerial occupation (53.0%) had early ANC, with those into Agriculture (36.9%) being the least to initiate ANC early. The prevalence of early initiation was similar for working women of all ages though slightly higher for those in 25–29 age bracket (41.2%). Those with higher education dominated in early ANC initiation (54.1%). Cohabiting women had the highest proportion of ANC visits (44.9%) just as Traditionalists (41.7%). The richest (50.5%) as well as urban residents (45.9%) had high early commencement of ANC visits. The results indicated that 44.5% of women at parity one initiated ANC visits early. Again, 41.6% of women who considered their pregnancy as a planned one had early initiation of ANC visits, just as women whose household heads were females (42.1%).

Additionally, 72.3% and 57.2% of women who read newspaper/magazine and watched television almost every day respectively, initiated ANC visits early. In the same vein, those that listened to radio almost every day (55.2%) dominated in early initiation of ANC visits. Women who considered getting permission to utilise existing medical assistance as not a problem were the highest (47.1%) to commence early ANC visits. In a similar manner, those that felt getting money needed for seeking medical treatment is not a problem (44.4%) as well as women that considered distance to health facility as not a problem (48.7%) topped early initiation of ANC visits. From the chi-square tests, type of occupation and the control variables were statistically significantly associated with early initiation of ANC, as can be seen from Table 2.

### Logistic regression results

Table 3 displays the inferential results of the study. Compared to managerial workers, women in all other work categories had lower odds of early ANC initiation and this was phenomenal among agricultural workers [cOR = 0.53, CI = 0.51, 0.56] and this continued after adjusting for the covariates [aOR = 0.74, CI = 0.69, 0.79]. Women from Liberia [aOR = 3.14, CI = 2.84,3.48] and Comoros [aOR = 2.56, CI = 2.14,3.07] had higher tendency of early ANC visits compared with those from Angola. Women aged 35–39[aOR = 1.38, CI = 1.28,1.48], and 40-44 [aOR = 1.38, CI = 1.28,1.50], were most inclined to early initiation of ANC visits as compared with those aged 15–19 just as among those with higher education as compared with those with no formal education [aOR = 1.49, CI = 1.37,1.61]. The analysis also revealed that the married women had a higher propensity of early ANC visits as compared to the never married [aOR = 1.30, CI = 1.23,1.38]. It was evident that the likelihood of early ANC visits among women decreased across religions especially among those who were not affiliated to any religion as compared to the Christians [aOR = 0.80, CI = 0.74,0.87]. The richest were most probable to commence ANC early as compared with the poorest [aOR = 1.44, CI = 1.36,1.51]. Furthermore, the rural residents were more likely to start ANC early as compared with the urban residents [aOR = 1.08, CI = 1.04,1.12]. Moreover, the probability to initiate ANC visit early declined among women at various parities especially among those at parity four or more compared with those at parity one [aOR = 0.67, CI = 0.63,0.70], as can be seen from Table 3.

**Table 3:** Logistic regression analysis on women’s occupation and early ANC visit in SSA

**Type of occupation **	Model I cOR[95%CI]	Model II aOR [95%CI]
Managerial	1 [1.00,1.00]	1 [1.00,1.00]
Clerical	0.88^*^[0.77,0.99]	0.91[0.80,1.04]
Sales	0.64^***^[0.61,0.68]	0.83^***^[0.78,0.88]
Agriculture	0.53^***^[0.51,0.56]	0.74^***^[0.69,0.79]
Services	0.55^***^[0.52,0.59]	0.88^***^[0.82,0.94]
Manual	0.62^***^[0.58,0.66]	0.77^***^[0.72,0.83]
**Age**		
15-19		1 [1.00,1.00]
20-24		1.13^***^[1.07,1.20]
25-29		1.29^***^[1.21,1.38]
30-34		1.35^***^[1.26,1.45]
35-39		1.38^***^[1.28,1.48]
40-44		1.38^***^[1.28,1.50]
45-49		1.37^***^[1.24,1.51]
**Level of education**		
No formal education		1 [1.00,1.00]
Primary		1.16^***^[1.12,1.20]
Secondary		1.23^***^[1.18,1.28]
Higher		1.49^***^[1.37,1.61]
**Marital status**		
Never married		1 [1.00,1.00]
Married		1.30^***^[1.23,1.38]
Cohabitation		1.18^***^[1.11,1.26]
Widowed		1.20^**^[1.07,1.33]
Divorced		1.25^***^[1.16,1.34]
**Religion**		
Christianity		1 [1.00,1.00]
Islam		0.88^***^[0.85,0.92]
Traditional		0.86^***^[0.80,0.92]
No religion		0.80^***^[0.74,0.87]
**Wealth quintile **		
Poorest		1 [1.00,1.00]
Poorer		1.02[0.98,1.05]
Middle		1.05^**^[1.01,1.09]
Richer		1.13^***^[1.08,1.17]
Richest		1.44^***^[1.36,1.51]
**Residence **		
Urban		1 [1.00,1.00]
Rural		1.08^***^[1.04,1.12]
**Parity**		
1		1 [1.00,1.00]
2		0.87^***^[0.83,0.90]
3		0.79^***^[0.76,0.83]
4+		0.67^***^[0.63,0.70]
**Pregnancy intentions**		
Planned		1 [1.00,1.00]
Mistimed		0.81^***^[0.78,0.83]
Unwanted		0.78^***^[0.74,0.82]
**Household head’s sex**		
Male		1 [1.00,1.00]
Female		1.09^***^[1.05,1.12]
**Frequency of reading newspaper/magazine**		
Not at all		1 [1.00,1.00]
Less than once a week		1.04[0.99,1.09]
At least once a week		1.11^***^[1.05,1.18]
Almost every day		1.25^**^[1.06,1.47]
**Frequency of watching television**		
Not at all		1 [1.00,1.00]
Less than once a week		0.95^**^[0.91,0.99]
At least once a week		1.07^***^[1.03,1.11]
Almost every day		1.27^***^[1.18,1.36]
**Frequency of listening to radio**		
Not at all		1 [1.00,1.00]
Less than once a week		0.99[0.96,1.03]
At least once a week		1.03[1.00,1.06]
Almost every day		1.09^*^[1.01,1.17]
**Getting medical help for self: getting permission to go**		
No problem		1 [1.00,1.00]
Big problem		0.93[0.83,1.04]
Not a big problem		0.92[0.82,1.03]
**Getting medical help for self: getting money needed for treatment**		
No problem		1 [1.00,1.00]
Big problem		1.27^**^[1.07,1.50]
Not a big problem		1.33^***^[1.12,1.57]
**Getting medical help for self: distance to health facility**		
No problem		1 [1.00,1.00]
Big problem		0.98[0.87,1.10]
Not a big problem		1.01[0.89,1.13]
**Country **		
Angola		1 [1.00,1.00]
Burkina		1.09[1.00,1.19]
Benin		1.78^***^[1.63,1.93]
Burundi		1.18^***^[1.09,1.29]
Congo DR		0.29^***^[0.27,0.32]
Congo		1.24^***^[1.13,1.35]
Cote d’Ivoire		0.66^***^[0.60,0.73]
Cameroon		0.74^***^[0.67,0.82]
Ethiopia		0.74^***^[0.66,0.83]
Gabon		1.66^***^[1.48,1.87]
Ghana		2.49^***^[2.25,2.75]
Gambia		0.99[0.88,1.10]
Guinea		0.70^***^[0.63,0.78]
Kenya		0.25^***^[0.22,0.28]
Comoros		2.56^***^[2.14,3.07]
Liberia		3.14^***^[2.84,3.48]
Lesotho		0.83^*^[0.71,0.96]
Mali		1.13^*^[1.01,1.26]
Malawi		0.43^***^[0.40,0.47]
Nigeria		0.37^***^[0.34,0.40]
Namibia		0.94[0.82,1.06]
Rwanda		1.65^***^[1.51,1.81]
Sierra Leone		1.26^***^[1.12,1.41]
Senegal		2.55^***^[2.31,2.81]
Chad		0.96[0.84,1.10]
Togo		0.45^***^[0.41,0.50]
Uganda		0.55^***^[0.51,0.60]
Zambia		0.37^***^[0.33,0.40]
Zimbabwe		0.66^***^[0.59,0.73]
*N*	131912	131912

Exponentiated coefficients; 95% confidence intervals in brackets

^*^ *p* < 0.05, ^**^ *p* < 0.01, ^***^ *p* < 0.001

Furthermore, those that considered their pregnancies as unwanted were least likely to have early ANC visit compared with those that judged theirs as planned [aOR = 0.78, CI = 0.74,0.82]. The propensity to early ANC initiation was higher among women whose household head was a female compared with those having male as household head [aOR = 1.09, CI = 1.05,1.12]. Also, those that read newspaper/magazine almost every day were most likely to have early ANC visit as compared with those that do not read newspaper/magazine at all [aOR = 1.25, CI = 1.06,1.47]. Those that watched television almost every day were inclined to early ANC initiations compared to their counterparts who did not watch television at all [aOR = 1.27, CI = 1.18,1.36]. Similarly, women that listened to radio almost every day were most likely to commence ANC early compared with those who did not listen to radio at all [aOR = 1.09, CI = 1.01,1.17]. Finally, the results indicated that those that judged that getting money needed for treatment was not a big problem were most likely to initiate ANC early than those that considered it as no problem [aOR = 1.33, CI = 1.12,1.57], as shown in Table 3.

## Discussion

Despite the scale-up of ANC services in SSA, a growing body of knowledge shows different level of adoption [4, 10]. Hence, the focus of this study was to investigate maternal occupational type and early ANC initiation in SSA. Our findings show that Liberia had the highest early ANC visit while DR Congo had the least. This finding is in line with a prior study in Ethiopia, where a prevalence of was recorded [35], Nigeria [19], and Ethiopia [17]. However, prevalence of our study was lower compared to Gulema et al. [36] study in Ethiopia, Paudel, Jha, and Mehata [34] study in Nepal and Moller et al. [10] study among women in developed countries. The inconsistency in the findings could be due to the scope, time gap and other methodological variations between our study and the other studies compared. We also observed regional variations in early ANC visits, which could be due to difference in women socioeconomic status, government health policies, and different cultural orientations within SSA.

We observed a trend of increasing early ANC attendance in recent times. The rise in early ANC visits within SSA may be due to some initiatives by governments of SSA countries. For instance, WHO [1] reports that tremendous investment has been made in the Liberian healthcare sector by stakeholder organisations and the government after the civil war. These investments might have helped improved maternal healthcare services leading to high ANC initiation as observed in this study [37]. Similarly, the Government of Ghana in 2005 initiated a maternal health policy with free maternal health services at ANC [24] meant to remove economic inequalities especially for rural women. Where such similar healthcare policies exist such as Nigeria [38], Tanzania [39] and Kenya [40] increased early ANC more probable. It is therefore our advocacy that governments and policy makers within the sub region consider eliminating all forms of economic, sociocultural, political and structural barriers that might impede women’s access and utilisation of early ANC services.

Also, women from Liberia and Comoros had higher tendency to commence early ANC visits compared with those from Angola. The reasons for such disparities cannot possibly be fathomed without further investigation. Yet it is sound to reason that Liberia and Comoros could have enhanced facilitators to maternal ANC utilisation incentives coupled with limited barriers to maternal ANC services utilisation. For instance, a report by Agence Francaise De Development [AFD] [41] show the Government of Comoros and its development partner France has consistently over the period invested heavily in the healthcare sector to increase access to health facilities, reduce financial barriers by providing financial incentives for obstetric care and many maternal health services. This according to the same report has led to the decrease in infant and maternal mortalities in Comoros and increase ANC utilisation among others. Such opportunity may be absent in other sub regional countries, and could be the driving force for early ANC visit in Comoros. We suggest to other SSA governments and policy analysts to under study these two countries for best practices that are influencing early maternal ANC visits.

Besides, our findings showed that women in all other work categories had lower odds of early ANC initiation and this was phenomenal among agricultural workers. A possible inference is that women in managerial positions have much more enablers to early ANC visit. This is possible because managerial mothers may have acquired the necessary educational and economic empowerments which is observed in previous studies to be associated with early ANC [24]. This finding is consistent with the National Survey analysis by Saad-Haddad et al. [42] that mothers’ occupational status significantly affects maternal early ANC visits. Implications of this finding is that, despite the introduction of the free maternal health policy in some SSA countries to enable mothers meet the recommended WHO early ANC visit, there are s still challenges partly influenced by monetary capabilities where highly placed occupational mothers can access maternal health services compared to other occupational types [22, 24]. This has been observed in our finding where mothers who revealed that getting money needed for treatment was not a big problem and richest mothers were most likely to have early ANC visit. This corroborates Akowuah et al. [23] and Arthur’s [24] findings that wealth significantly influences early ANC visit among mothers. It is thus imperative for SSA countries without social interventions that can enhance early ANC visit to introduce social interventions and provide sustainable jobs to women so as to remove financial barriers to maternal health service utilisation.

Again, our findings show that women aged 40–44 were inclined to early ANC visits compared to aged 15–19. Inferring from Grossman model of prediction, which states that age increases the rate of depreciation of one’s health [43], that is, older mothers may be prone to certain health conditions including pregnancy complications, and may therefore be motivated to initiate early ANC. Similarly, evidence from a qualitative study by Pell et al. [25], in Ghana, Kenya and Malawi support our finding that younger adolescents do not initiate early ANC as recommended by WHO compared to older adults. Additionally, other quantitative studies in Ethiopia [3], Finland [44] and India [45] add credence to our finding that older women initiate early ANC compared to younger mothers. We reasoned with Pell et al. [25] that, due to social stigma and repercussions of teen pregnancy (i.e., drop out from school), adolescents are hesitant in disclosing pregnancy and may hinder early ANC initiation. This calls for segregated adolescent friendly ANC clinics to improve early maternal ANC among adolescents, which consequently may help reduce morbidities and mortalities among adolescent mothers and their new-borns. On the contrary, some scholars indicate that younger mothers reported early ANC utilisation in discordance with our findings [3, 24, 46]. The difference in the findings could be due to some methodological variations such as sample size differentials. We suggest that SSA governments and policy makers be critical on age demography, especially in maternal ANC services provision in order to meet sustainable development goal 3 and 5 [47].

Women who considered their pregnancies as unwanted were least likely to early initiate ANC compared with those that judged theirs as planned. This finding resonates previous studies [19, 48, 49]. In a similar finding, Alemu and Aragaw [19] adduced that a woman who is yearning for a child would likely initiate early ANC once diagnosed of being pregnant. However, a woman carrying an unwanted pregnancy might already be contemplating on terminating the pregnancy and so would not see the need to engage in early ANC to protect the gestation of something she does not want [50].

The propensity to initiate early ANC was higher among women whose household head was a female compared with those having a male as household head. Essentially, for a pregnant woman to seek permission from someone else especially the opposite sex, is a disincentive to early ANC initiation [51]. So, for women to access and obtain optimum health at ANC, their independent decision-making may be essential.

Our findings revealed that mothers with higher education, those who resides in rural areas, those who were exposed to mass media (i.e., radio, reading newspaper/magazine and watching television daily) were likely to initiate early ANC. Arthur [24] similarly found that women who had higher education, and women who listen to radio and watches television daily had early ANC visit. It is possible that most non-educated mothers may reside in rural areas which are mostly deprived of health facilities and the right tailored media information [52]. Although women in urban locations may have better geographical access to health facilities compared with rural residents, hectic traffic and heavy workloads could compromise the ability of urban women from having early ANC. Notwithstanding, a systematic review on rural residence and early ANC initiation concluded inconsistent with our finding [53] which could be due to contextual factors.

Another significant finding of our study was the propensity of married women to initiate early ANC compared to the never married. This could be reasoned that the married women may have their partners support (i.e. financial and psychosocial) which may not be available for the never married. Community action is needed to encourage men participation in ANC. Moreover, the probability of early ANC visit declined among women at various parities especially among those at parity four or more compared with those at parity one. This finding was probably expected as previous studies in SSA [3] and elsewhere [54], showed that high parity mothers exhibited late ANC visit. This finding could be influenced by maternal experience, complacency, and pregnancy risk assessment which could have far reaching consequences for both mother and fetus. Women with four or higher parity have increased risk of haemorrhage, however, most of these women are not educated on the need to prioritise ANC and subsequent health facility delivery, where blood transfusion can transpire [55, 56]. Because of this, most of these women, would erroneously perceive that they are not at risk since they had no problems in earlier pregnancies. Healthcare providers must therefore consistently educate and remind women of higher parity on the need to prioritise ANC and health facility delivery, due to their higher risk of experiencing haemorrhage and other childbirth complications.

### Strengths and limitations of the study

Findings from this study were generated from high quality data gathered through the DHS Program. The sampling procedure and representativeness of the datasets strengthen the generalisability of the findings. Conversely, the cross-sectional design of the DHS limits the ability to make causal inferences between the outcome variables, however, associations can be drawn between occupation type and early ANC visit. The study presented evidence at the sub-regional level, thereby making recommendations not directly applicable at the national level. Due to this, we mentioned the specific countries in instances of extreme cases and we have also indicated in the conclusion section that implementation of our recommendations should be guided by contextual circumstances per country. Some of the variables were also self-reported, hence, there is a high tendency for social desirability and recall biases. We recommend that further studies should employ qualitative study designs and theoretical models for a better understanding of nuances surrounding type of occupation and early ANC attendance.

## Conclusion

The findings show that women in managerial and clerical occupations are most likely to initiate ANC early, however, the opposite was true for women in Agricultural activities. Generally, early ANC visit is phenomenal among women of Liberia whilst DR Congo lags behind. These observations bring to bear some essential elements worth considering to enhance early ANC visits within SSA irrespective of occupation type. First, it is time to review health insurance policies across SSA to incorporate measures that can help absorb the additional cost that are currently outside the remit of the insurance policies for maternity care. Second, women in agricultural industry need much more attention in order to bridge the early ANC visit gap between them and workers of other sectors or industries. Third, critical review of the maternal health service delivery of DR Congo is needed considering the low rate of early ANC visits. A qualitative study is essential to gain a better understanding of the enablers of early ANC visits among women in managerial roles as well as barriers that compromise early ANC among agricultural workers. These recommendations should be considered with respect to country-specific contextual factors, considering the different cultural orientations, political structures and varied health systems within SSA.

## Data Availability

The datasets supporting the conclusions of this article are available in the Measure DHS repository, https://dhsprogram.com/data/available-datasets.cfm.

## Abbreviations

AFD: Agence Francaise De Development
ANC: Antenatal care
aOR: Adjusted Odds Ratio
CI: Confidence Interval
cOR: Crude Odds Ratios
DHS: Demographic and Health Survey
MMR: Maternal mortality ratio
SSA: Sub-Saharan Africa
WHO: World Health Organisation
